# Targeting WNT Signaling for Multifaceted Glioblastoma Therapy

**DOI:** 10.3389/fncel.2017.00318

**Published:** 2017-10-13

**Authors:** Matthew McCord, Yoh-suke Mukouyama, Mark R. Gilbert, Sadhana Jackson

**Affiliations:** ^1^Neuro-Oncology Branch, National Cancer Institute, Bethesda, MD, United States; ^2^Laboratory of Stem Cell and Neuro-Vascular Biology, Genetic and Developmental Biology Center, National Heart, Lung and Blood Institute, Bethesda, MD, United States

**Keywords:** WNT, glioblastoma, drug delivery, angiogenesis, cancer biology

## Abstract

The WNT signaling pathway has been of great interest to developmental biologists for decades and has more recently become a central topic for study in cancer biology. It is vital for cell growth and regulation of embryogenesis in many organ systems, particularly the CNS and its associated vasculature. We summarize the role of WNT in CNS development and describe how WNT signaling makes key contributions to malignant glioma stemness, invasiveness, therapeutic resistance, and angiogenesis. The role of WNT in these mechanisms, along with creation and maintainance of the blood-brain barrier (BBB), points to the potential of WNT as a multi-faceted target in malignant glioma therapy.

## Introduction

The WNT signaling pathway is one of the most heavily studied in cell biology; it influences many processes in embryonic development, physiology, and homeostasis. The gene now known as WNT was first described by Nusse and Varmus (1982) in mouse mammary tumors; it was dubbed Integration 1 (Int1). Five years later, it was recognized as the mammalian equivalent of a Drosophila gene associated with a wingless phenotype (Wg). Over the next decade, the signaling pathway associated with the protein was further defined. The role of WNT signaling in embryonic development, particularly in axis patterning and the differentiation of anterior and posterior CNS structures, was elucidated. Abnormalities in WNT signaling are associated with many pathologies in multiple organ systems, including the nervous system. Specifically, it has been associated with congenital defects of multiple brain structures, including the cerebellum, midbrain, and thalamus (McMahon and Bradley, 1990; Thomas and Capecchi, 1990; Zhou et al., 2004). More recently, research has begun to define the role of WNT signaling in CNS tumors (Klaus and Birchmeier, 2008).

Glioblastoma is the most common primary CNS malignancy. Rapid cell proliferation, treatment resistance, and abundant angiogenesis characterize these aggressive tumors. Despite advances in surgical techniques, radiation therapy, and better understanding of tumor biology, the median surival remains <18 months. Many newer agents, with varied treatment mechanisms, have failed in clinical studies. Clearly, better therapies that collectively target different aspects of glioma pathogenesis are needed.

There is growing interest in the potential role of WNT signaling in malignant glioma pathogenesis, and how it could be targeted therapeutically. For example, FAT atypical cadherin 1 (FAT1) and the hepatocyte growth factor (HGF) pathway are both known molecular features of glioma pathogenesis. Intriguingly, both appear to be connected to WNT signaling (Birchmeier et al., 2003; Kong et al., 2009; Kim et al., 2013; Morris et al., 2013). A number of molecular pathways are believed to contribute to specific features of glioma biology, through crosstalk with WNT signaling. These include Pleomorphic adenoma gene-like 2 (PLAGL-2) associated with glioma stem cells; SNAIL gene, which promotes epithelial-mesenchymal transition (EMT) and tumor invasiveness; Frizzled-1 (FZD1), associated with radiation resistance; and vascular endothelial growth factor (VEGF), which promotes angiogenesis and vasculogenesis (Zheng et al., 2010; Jin et al., 2011; Liu et al., 2015).

Malignant gliomas present a therapeutic challenge; they are protected from cytotoxic chemotherapy by a heterogeneously permeable blood-brain barrier (BBB). Since WNT plays an essential role in development of CNS vasculature and establishment of key structural and functional BBB features, targeting WNT to improve BBB permeability and drug delivery is a plausible consideration (Liebner et al., 2008; Stenman et al., 2008; Daneman et al., 2009).

In this review, we first outline the structure and biochemistry of WNT and associated proteins. We go on to summarize the role of WNT in development of the CNS and associated vasculature. We then describe the growing body of evidence for the contribution of WNT signaling in malignant glioma, with emphasis on key features of tumor biology. Finally, we discuss how WNT-based therapies present an opportunity to target not only the tumor, but also associated microvasculature to improve drug delivery and possibly attenuate angiogenesis.

## Structural and functional features of the WNT pathway

There are at least 19 members of the WNT family. WNT proteins are rich in cysteine, and have a highly conserved cysteine sequence (Kikuchi et al., 2011). WNTs are approximately 350 residues in length (Logan and Nusse, 2004). The N-terminal domain consists of a group of alpha-helices. The C-terminal domain is characterized by two beta sheets, also joined by disulfide bridges (Willert and Nusse, 2012). Several WNT proteins are post-translationally glycosylated, a process which seems to be important for their secretion (Smolich et al., 1993; Kurayoshi et al., 2007). Depending on the individual WNT, lipidation may or may not be necessary for either secretion or receptor binding (Kikuchi et al., 2011). WNTs tend to be quite hydrophobic, and a number of them undergro post-ranslational lipidation with palmitate and/or palmitoleic acid at various residues.

**Figure 1:**
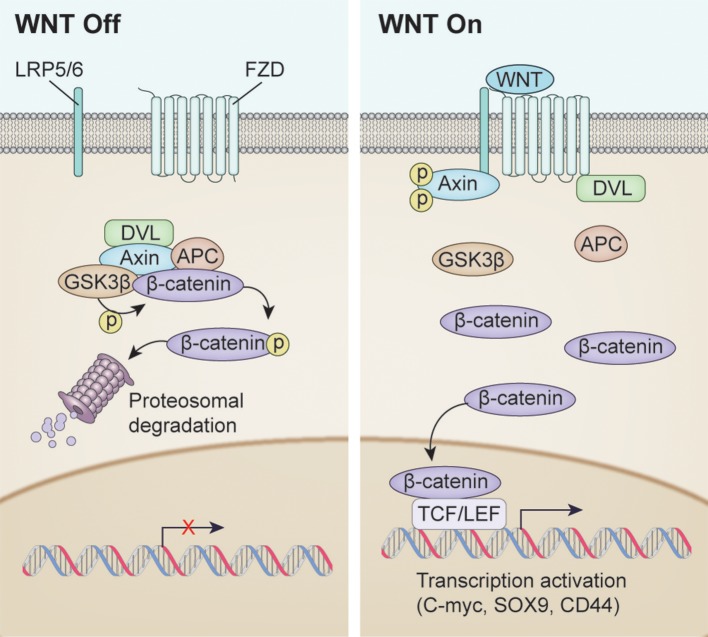
Overview of WNT signaling pathway activation. **(Left)** WNT signaling inactivation with absence of the WNT ligand. Phosphorylation of β-catenin resulted in Dishevelled (DVL), Axin, APC and GSK3β complex resulting in proteosomal degradation. **(Right)** Canonical WNT signaling activation after WNT ligand binding. Unphosphorylated β-catenin enters the nucleus to drive transcription affecting such genes as C-MYC, SOX9, CD44.

The WNT/β-catenin signaling pathway, also known as the canonical WNT pathway, is perhaps the best characterized (Figure 1). The protein β-catenin is a subunit of the cadherin complex, a group of proteins which form cellular junctions (McCrea et al., 1991). The central feature of the canonical WNT pathway is stabilization of cytosolic β-catenin followed by translocation to the nucleus (Niehrs, 2012). Normally, β-catenin is found in the cytosol. However, when β-catenin accumulates in the nucleus, it forms multimeric complexes with other transcription factors, including several members of the TCF family and Lef-1, to facilitate expression of multiple genes invovled in cell proliferation including c-myc, n-myc, Sox9, and CD44 (He et al., 1999; Wielenga et al., 1999; Blache et al., 2004; Shu et al., 2005; Valenta et al., 2012).

β-catenin independent or “non-canonical” WNT signaling may be dividied into two pathways, the planar cell polarity (PCP) pathway and the WNT/calcium pathway (Gordon and Nusse, 2006; Komiya and Habas, 2008). The PCP pathway controls the polarization of epithelial cells along the plane of the basement membrane (Darken et al., 2002). The WNT-Calcium pathway mediates release of Ca^2+^ from the ER into the cytosol. It is involved in the control of embryonic dorsal axis formation, gastrulation, and tissue separation.

Three of the most important WNT family members, WNT3a, WNT5a, and WNT7a, function as ligands in the signaling pathways. WNT3a, the first to be biochemically isolated, is quite hydrophobic due to lipid modification. WNT3a is an active ligand in β-catenin signaling, and also plays a role in stimulating the growth of hematopoietic stem cells (Willert et al., 2003; Samarzija et al., 2009). WNT5a plays a key role in non-canonical WNT signaling. It can either stimulate or inhibit the canonical β-catenin pathway. WNT5a has also been implicated in atherosclerosis and vascular thrombosis (Kim et al., 2011; Bhatt and Malgor, 2014). WNT7a can function as a ligand in both the canonical β-catenin pathway, and in the non-canonical PCP pathway. WNT7a is one of the most extensively studied WNT ligands, and one of the most functionally diverse. It can stimulate both canonical and non-canonical pathways (Carmon and Loose, 2008; Le Grand et al., 2009). Additionally, it plays key roles in embryogenesis, and in the pathogenesis of several types of cancer (Bui et al., 1997; Yoshioka et al., 2012; Bikkavilli et al., 2015).

## WNT signaling in development of the nervous system and associated vasculature

WNT signaling has been of great interest in developmental biology. We will briefly consider its role in neurodevelopment. WNT/β-catenin signaling is crucial for the formation of the primitive streak, a structure that develops in the posterior region of the embryo. Specifically, epiblast cells undergo epithelial-to-mesenchymal (EMT) transition, ingressing initially to give rise to the endoderm and mesoderm of the head and heart, and later to progressively more posterior mesoderm types, including somites (Liu et al., 1999; Mohamed et al., 2004). In mouse embryos, the anterior visceral endoderm (a defining feature of the rostral pole) expresses high levels of Dickkopf-1 (DKK1), a Wnt inhibitor which contributes to induction of cephalic development (Glinka et al., 1998). As we will consider later, local expression of paracrine WNT inhibitors, like DKK1, play a role in the oncogenesis of certain brain tumors, and also suggests a unique and fascinating potential therapeutic role for WNT inhibition.

Activity of the WNT/β-catenin pathway is vital for differentiation of the dorsal aspect of the spinal cord. WNT signaling, along with Sonic Hedgehog (SHH) signaling, facilitates dorsoventral polarization of the spinal cord in vertebrates. SHH serves as a morphogen and is expressed initially in the notochord, and later in the ventral cells of the neural tube (Roelink et al., 1994; Marti et al., 1995). In conjunction with its receptor “patched” (Ptc), SHH directs patterning and morphogenesis of the ventral spinal cord (Stone et al., 1996). Similar to the pattern found in spinal cord development, a gradient of WNT signaling appears to direct the early differentation of anterior as opposed to posterior brain structures in many vertebrate species (McMahon and Bradley, 1990; Heisenberg et al., 2001; Ciani and Salinas, 2005). Experimentally disrupting normally-occuring WNT inhibitors, such as DKK1, results in posteriorization of the anterior embryonic brain, leading to effects, such as cyclopia (Kazanskaya et al., 2000; Mulligan and Cheyette, 2012). The WNT/β-catenin pathway is vital for regulation of dorsal-ventral patterning in the telencephalon, inducing a dorsal phenotype in cells (Gunhaga et al., 2003). Mutations in WNT's co-receptor, LRP-6, lead to hypoplasia of the dorsal thalamus, and lack of thalamocortical projections in mice (Zhou et al., 2004). Knockout of WNT3a leads to absence of the hippocampus in mice (Lee et al., 2000). The hippocampus also fails to develop in mouse models when WNT expression is transcriptionally disrupted in the cortical hem, a signaling center in the embryonic brain (Mulligan and Cheyette, 2012).

While WNT was initially investigated in the context of the nervous system for its role in developmental patterning of the brain and spinal cord, more recent studies have established the importance of WNT signaling in the growth and guidance of axons, synapses and neural circuits. In axons, the WNT signaling protein Dishevelled (DSV) interacts with microtubules and appears to play a role in cytoskeletal dynamics and stabilizes actin in axon microtubules, increases axon diameter and growth cone size (Ciani et al., 2004). In addition, DSV1 (a specific isoform of DSV) regulates the development of hippocampal dendrites in mice (Rosso et al., 2005). Disruption of WNT7a, normally found in cerebellar granular cells, inhibits mossy fiber growth cone modulation. WNT7a not only modulates growth cone activity in the cerebellum, but also the formation of synapses between granular cells and mossy fibers (Hall et al., 2000).

Of particular relevance to our discussion is the role of WNT signaling in development of the CNS vasculature and BBB. Brain vasculature is unique in that it tightly restricts which molecules can penetrate the CNS parenchyma (Liebner et al., 2008; Zhou et al., 2014). In the developing spinal cord (neural tube), angioblasts and endothelial cells are recruited and coalesce into the peri-neural vessel plexus (PNVP) on the surface of the neural tube. As development proceeds within the neural tube, blood vessels invade the neuroectoderm via sprouting angiogenesis—forming an intra-neural vessel plexus (INVP). In both the PNVP and INVP, endothelial cells begin to develop structural associations with astrocyte foot processes, pericytes, and neurons. Molecular cross-talk between the cell groups also begins to occur (Bautch and James, 2009).

Tight junctions amongst CNS endothelial cells significantly restrict the permeability of the BBB, preventing many substances from crossing the vascular lumen. The claudins (most prominently claudin-1, claudin-3, and claudin-5) are one of the families of proteins which make up tight junctions, and WNT signaling contributes to their activity (Findley and Koval, 2009). Specifically, β-catenin signaling induces expression of claudin-3, while experimental knockdown of WNT signaling in the embryonic brain results in absence of normal cell-cell junctional organization. This leads to vessels which are abnormally enlarged, leaky, and hemorrhagic. This abnormal phenotype is associated with elevated expression of plasmalemma vesicle associated protein (PLVAP) (Liebner et al., 2008). PLVAP is a key structural component of capillary fenestrations. It facilitates normal vascular physiology in organs with permeable capillaries, such as in kidneys and endocrine organs, but is not normally found in brain vasculature (Satchell and Braet, 2009; Guo et al., 2016; Phoenix et al., 2016). The role of WNT signaling in BBB development and its potential to influence vascular permeability is central to our discussion of the pathway as a therapeutic target in CNS tumors.

CNS-specific deletion of *wnt7a* and *wnt7b* as well as endothelial cell-specific deletion of ß-catenin in mice, resulted in severe CNS-specific hemorrhage due to dilated PNVP and defective INVP endothelial cells and pericytes in the neural tube. These results indicate that canonical Wnt signaling in endothelial cells is important for PNVP integrity and blood vessel ingression to form the INVP (McCrea et al., 1991; Gordon and Nusse, 2006; Komiya and Habas, 2008; Niehrs, 2012). CNS-specific hemorrhage observed in tumors with these mutations highlights the role of canonical Wnt signaling in the BBB formation. The BBB has a number of components, including endothelial cells linked together by tight and adherens junctions, pericytes, astrocytic foot processes, and efflux pumps. Additionally, pericyte recruitment is essential for stabilization of the BBB and for maintenance of tight junctions. WNT7a and 7b facilitate development of BBB characteristics in CNS endothelial cells based on the induction of the glucose-transport protein Glut1 (McCrea et al., 1991; Niehrs, 2012). The endothelial G-protein coupled receptor (GPCR) Grp124 specifically enhances WNT7a and 7b-mediated canonical signaling to control CNS angiogenesis and BBB permeability (Anderson et al., 2011; Zhou and Nathans, 2014; Posokhova et al., 2015; Vanhollebeke et al., 2015; Chang et al., 2017).

The role of WNT signaling in the pathologic neovascularization of retinal disease highlights its importance in maintenance of healthy CNS vasculature. One of the hallmarks of pathologic retinal neovascularization is disruption of the blood-retinal barrier, which results in significant vascular leakiness (Lobo et al., 2004; Schulenburg and Tsanaktsidis, 2004). Mouse models of hypoxic retinopathy demonstrate that components of the WNT signaling pathway, including LRP5, and increased expression of WNT ligands play a key role in the pathogenesis. Intriguingly, disruption of WNT signaling in the same models leads to a less severe retinopathy (Chen et al., 2011). An established role for WNT in certain forms of pathologic angiogenesis suggests a potential role for WNT inhibition in anti-angiogenesis therapy for malignancy.

## WNT signaling in malignant gliomas

WNT signaling is of growing interest in neurooncology research. The established role of WNT in the pathogenesis of a number of non-CNS and CNS malignancies points to the need for investigation of a potential role in malignant gliomas. WNT signaling is known to play a role in the pathogenesis of colorectal cancer, hepatocellular carcioma, and in one sub-type of medulloblastoma. Specific mechanisms of WNT signaling in these tumors will later be discussed in the context of tumor vasculature. As of yet, relatively few WNT pathway alterations have been identified in malignant gliomas. However, we will briefly consider those that have. FAT1 is a protocadherin family protein that binds β-catenin, inhibiting its activity as a transcription factor. It belongs to the protocadherin family, a group of transmembrane proteins found in epithelial tissues, which are believed to play a role in cell-cell interactions. FAT1 acts as a tumor suppessor by inhibiting cell cycle progression from G1 to S. This β-catenin translocation to the nucleus then increases transcriptional activation and cell growth. It is proposed that FAT1 function promotes dysregulated WNT/β-catenin signaling, allowing more free β-catenin to enter the nucleus. Recently, homozygous deletion of FAT1 was observed to occur in approximately 57% of glioblastomas and this deletion was associated with a significantly prolonged survival (Morris et al., 2013).

WNT is also connected to parallel signaling pathways involved in glioblastoma pathogenesis. HGF and its receptor c-Met have well-established roles in the pathogenesis of several human cancers, including hepatocellular carcinoma, colorectal cancer, and glioblastoma (Birchmeier et al., 2003). Expression of c-MET is associated with a poor prognosis in glioblastoma, with median survival of 11.7 months compared to14.3 months in patients with lack of c-Met tumor expression(Kong et al., 2009). WNT/β-catenin signaling is significantly up-regulated in glioma stem cells that express high levels of c-Met. A similar correlation between c-Met expression and WNT/β-catenin signaling was observed in mouse glioma xenografts. Preclinical studies demonstrated that inhibition of c-MET in GBM cells decreased the nuclear translocation of β-catenin (Kim et al., 2013). This evidence suggests overlap of the WNT pathway with other pathways in glioblastoma which are relevant to the biology and clinical course of the tumor. For example, a therapeutic WNT inhibitor could attenuate tumor activity through both the FAT1 surface receptor and the HGF signaling pathway.

Particular applications of WNT signaling as it relates to four central features of malignant glioma biology are worth summarizing. Specifically, WNT signaling in stemness, invasivenss, therapeutic resistance, and tumor angiogenesis will be considered. Each of these processes presents a potential target for WNT blockade in malignant glioma therapy.

### WNT signaling and stemness

Stemness is an important concept in cancer biology. Fundamentally, it describes the ability of cells to self-renew and proliferate with a limited differentation status; reflecting the potential to develop into multiple types of cells (Cai et al., 2004). Malignant cells classically possess this phenotype, reflecting that of normal stem cells (found in the bone marrow, skin, and gonads) which are crucial for homeostasis (Wong et al., 2008). Glioma stem cells are thought to be the precursors of a variety of malignant gliomas (Lathia et al., 2015). WNT signaling is believed to contribute to glioma stem cell proliferation and survival; potentially providing an opportunity for therapeutic targeting. The mechanisms by which WNT signaling is involved in tumor stem cell biology are complex. Pleiomorphic adenoma gene like-2 (PLAGL-2), overexpressed in glioblastomas, was found to activate the WNT/β-catenin pathway in neural stem cells and contributes to glioma stem cell self-renewal. Human LN215 glioma cells expressing PLAGL-2 demonstrated increased expression of the neural stem cell marker nestin. Additionally, induced expression of PLAGL-2 was associated with a decreased expression of maturity markers, and impaired differentiation in neural stem cells. Preclinical studies in mice with PLAGL-2 expressing glioma xenografts developed disseminated disease with a reduced median survival compared with those injected with vector control glioma xenografts. Perhaps most interesting, treatment of PLAGL-2 expressing gliomas with the WNT inhibitor DKK1 significantly reduced the effect of PLAGL-2 on neural stem cells, and partially restored their ability to differentiate and proliferate (Zheng et al., 2010). Human achaete-scute homolog (ASCL1), an essential transcription factor in neuronal differentiation is also essential for maintenance and propagation of GSCs by upstream regulation of the Wnt pathway. Specifically, it can repress DKK-1 in GSCs, thereby promoting WNT signaling and GSC survival (Rheinbay et al., 2013). Collectively, these findings clearly suggest an active role of WNT signaling in glioma stem cell biology.

### WNT signaling and invasiveness

WNT signaling is known to contribute to invasiveness and metastasis in many malignancies. It is often associated with up-regulation of Frizzled-4 (FZD4), a positive regulator for WNT. EMT is a well-recognized process in many malignancies, in which epithelial tissue undergoes specific genetic and biochemical alterations to resemble and behave like mesenchymal tissue. After undergoing EMT, cells have increased migratory capacity and become quite resistant to apoptosis. The process plays a key role in tumor invasiveness and metastasis (Kalluri and Weinberg, 2009). Although the CNS lacks epithelial tissue, there is a growing consensus that a constellation of molecular and phenotypic changes, which parallel EMT, contribute to tumor invasivenss in malignant gliomas. Primarly glioblastomas are known to express key molecular markers associated with EMT. Specific examples include osteonectin (bone), YKL-40 (cartilage), and TNC (myeloid tissue) (Tso et al., 2006). In addition, cell lines derived from glioblastoma express a number of cell surface receptors associated with mesenchymal stem cells, such as CD29, CD44, and CD90 (Lee et al., 2014). A number of genes which contribute to EMT in other tumors are strongly associated with invasive activity in glioma cells and tumors. Some of these genes include SNAIL, TWIST, and ZEB1 (Elias et al., 2005; Mikheeva et al., 2010; Myung et al., 2014).

WNT has shown promise in blocking EMT-associated changes in other tumor types, and it may also hold potential in this regard for malignant glioma treatment. FZD4 induces SNAIL expression, which controls the epithelial-mesenchymal transition process (EMT) in malignant glioma cells (Jin et al., 2011), a central feature of tumor invasiveness. Induction of WNT signaling, through overexpression of positive regulators, increases expression of genes associated with EMT in glioma cells (SNAIL, TWIST, and ZEB1) (Lee et al., 2016). Additionally, the use of a Wnt/β-catenin inhibitor XAV939 prevented glioma cell invasion and EMT. Furthermore, WNT5a stimulation can induce migration of glioblastoma cells though β-catenin independent signaling, and by stimulating the activity of cell surface dissocating endopeptidase matrix metalloprotease 2 (MMP2). Accordingly, knockdown of WNT5a in human glioma cell lines suppressed cell invasion and migration by specifically decreasing MMP2 expression (Kamino et al., 2011). Thus, demonstrating the strong relationship between WNT inhibition in glioma cells to prevention of EMT and associated metastases.

### WNT signaling and therapeutic resistance

WNT signaling has been reported to contribute to temozolomide chemotherapy resistance in glioblasoma. *In vitro* studies evaluating glioma cells resistance to temozolomide, demonstrated overexpression of the developmental pathway of FZD2 (involved in Wnt pathway), and downregulation of transcription factor LEF1 (Wnt pathway inhibitor) (Auger et al., 2006).

Additionally, WNT signaling has been implicated as one of the culprits inducing radiation resistance in breast cancer. While radiation treatment kills murine mammary epithelial cells, radiation-resistant epithelial progenitor cells continued to proliferate. When these stem cells were analyzed further, they were found to have high levels of WNT/β-catenin activity (Woodward et al., 2007). In examination of glioblastoma cells with high levels of radiotherapy resistance, they also overexpress genes associated with WNT signaling. Genome analysis was performed on tumor cells from human xenograft models treated with whole brain irradiation (10Gy). Cells from recurrent tumors post-radiation had higher levels of activated β-catenin compared to cells from tumors after mock radiation. There was also increased expression of many genes associated with WNT signaling, including WISP1, FZD1, and APC. In contrast, post-radiation glioma cells exhibited much higher radiation sensitivity when irradiated after treatment with the WNT inhibitor XAV939 than did post-radiation glioma cells irradiated in the presence of a vehicle control (Kim et al., 2012).

### WNT signaling and tumor angiogenesis

WNT signaling is known to be involved in angiogenesis in a number of solid tumors (Sherwood, 2015). WNT regulates expression of VEGF, a key pro-angiogenesis factor in many types of cancer, including malignant gliomas (Zhang et al., 2001; Reardon et al., 2008; Liu et al., 2015). A detailed study of WNT, metabolism, and angiogenesis demonstrated that WNT/β-catenin signaling increases cytosolic lactate levels through increased aerobic glycolysis and pyruvate oxidation in colorectal cancer cells. An accompanying increase in monocarboxylate transporter-1 (MCT-1), a lactate transporter, causes increased lactate secretion promoting angiogenesis. WNT blockade in mouse colorectal tumor models resulted in signifcantly reduced tumor vascularity, and inhibition of tumor growth (Pate et al., 2014).

Studies with mouse tumor models of hepatocellular carcinoma (HCC) have shown that administration of WNT inhibitors (WIF-1, sFRP1) reduces the density of tumor vasculature, endothelial progenitor migration, and expression of pro-angiogenesis factors. Furthermore, the reduction in angiogenesis is associated with slower tumor growth and prolonged survival (Hu et al., 2009). WNT signaling has not been conclusively linked with angiogenesis in human gliomas, but its involvement in angiogenesis in other solid tumors, and particularly its relationship with VEGF, a known angiogenic factor in glioblastoma, strongly supports a connection. Thus, further studies evaluating the influence of WNT inhibition in glioma cells on tumor microvascular angiogenesis, and vasular growth factor expression are warranted.

### WNT signaling and drug delivery

The most prominent roles of WNT in cell function are related to cell proliferation and vascular stability. The WNT subtype medulloblastoma carries a WNT mutation which promotes abnormal nuclear β-catenin localization, inducing abnormal cellular proliferation (Baryawno et al., 2010). Intriguingly, this group has the best prognosis of all medulloblastomas, and responds exceptionally well to chemotherapy and radiation, even when diagnosed at an advanced stage (Northcott et al., 2011). The gene for β-catenin (CTNNB1) is frequently mutated in WNT medulloblastomas (Ellison et al., 2011). Other mutations specific to this tumor type include APC, AXIN1, and AXIN2 (DeSouza et al., 2014).

In 2016, the Phoenix research group published a landmark study that elucidated the vascular phenotype of WNT medulloblastomas (Phoenix et al., 2016). They reported markedly decreased expression of molecules normally associated with the brain vasculature, such as claudin-5 and Glut1. Additionally, plasmalemma vesicle-asociated protein (PLVAP, aka PV-1), a fenestration-specific protein, was conversely up-regulated. The presence of fenestrations, which is highly unusual in normal brain microvasculature, was also documented by electron microscopy. Furthermore, studies with rodent models demonstrated that WNT medulloblastoma xenografts have a much more favorable response to vincristine than SHH medulloblastoma xenografts. This is likely explained by the characteriscally poor BBB permeability of vincristine, a drug with good efficacy against medulloblastoma cells. However, pharmacodynamic experiments demonstrated that vincristine permeability was significantly higher in WNT medulloblastoma tumors, likely contributing to the improved outcomes of patients with this subtype.

Importantly, the same mutation in CTNNB1 that drives oncogenic WNT signaling and tumorigenesis in WNT medulloblastomas also induces secretion of locally active WNT inhibitors, such as WIF1 and DKK1. Experimental disruption of the observed paracrine signaling in tumor models results in restoration of the BBB and normalization of vascular permeability. Additional studies have demonstrated the connection between WNT signaling and PLVAP. Specifically, experimental knockout of β-catenin in mice leads to increased expression of PLVAP, and increased BBB permeability. These findings were confirmed by Evans Blue CNS tracer dye studies (Liebner et al., 2008). Similarly, mouse embryos with genetic knockout of the WNT signaling components LRP5 and LRP6 demonstrate increased levels of PLVAP and abnormal BBB leakiness (Zhou et al., 2014). These findings are fascinating, and suggest the possibility of targeting WNT sigaling to attenuate the BBB and improve delivery of chemotherapy drugs.

## WNT as a multi-faceted therapeutic target in brain tumors

As previously discussed, there is a growing body of evidence that WNT signaling plays a key role in malignant glioma pathogenesis, and contributes specifically to the growth of glioma stem cells, tumor invasiveness, and therapeutic resistance. A number of studies have shown notable effects from WNT modulation and/or inhibition in human cancers. Use of a small molecule WNT/β-catenin inhibitor, SEN461, demonstrated decreased glioma cell viability and subcutaneous implanted xenograft tumor volume (De Robertis et al., 2013). Additionally, Non-steroidal antiinflammatorys (NSAIDs) are being evaluated in clinical studies as potential therapeutic WNT inhibitors. Studies are ongoing for the effect of aspirin in esophageal and colorectal cancer, diclofenac in basal cell carcinoma and breast cancer, and celecoxib in pancreatic cancer and glioblastoma (Stockhammer et al., 2010; Brinkhuizen et al., 2016; Lee et al., 2016). Aspirin, thought to antagonize WNT by phosphorylating key residues on β-catenin and promoting its degradation, has been the most rigorously studied of the NSAID family for its anti-cancer properties. A retrospective clinical study of celecoxib (COX2-selective NSAID) in recurrent glioblastoma found that 6 month progression-free survival was 43% for patients receiving low-dose temozolomide plus celecoxib, as opposed to the 21% typically seen with standard temozolomide maintenance therapy. Median progression-free survival was 4.2 months in the celecoxib/temozolomide group (Stockhammer et al., 2010). Compelling evidence for WNT involvement in glioma pathogenesis combined with encouraging preliminary clinical results immediately suggests the value of a prospective, randomized trial of WNT inhibition (combined with standard chemotherapy plus radiaton) for glioblastoma patients.

**Figure 2:**
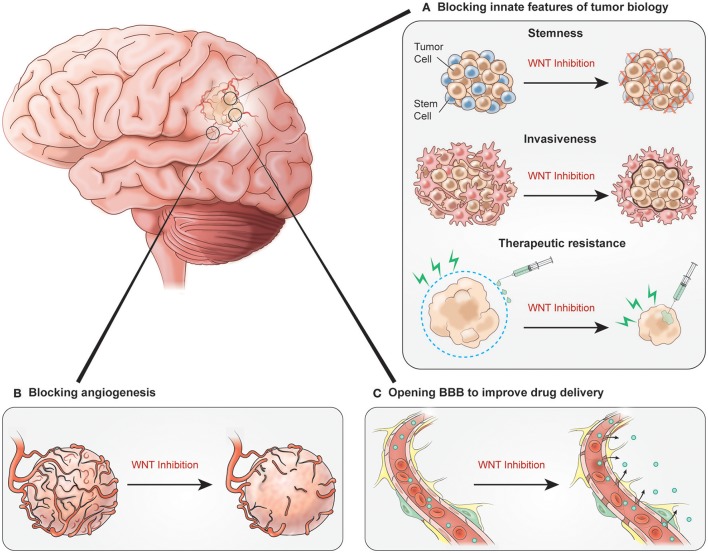
Potential benefits of inhibiting WNT signaling. **(A)** WNT inhibition blocking innate features of tumor biology could decrease tumor cell stemness, prevent tumor invasiveness and decrease therapeutic resistance. **(B)** Inhibition impacts glioblastoma vasculature by blocking angiogenesis. **(C)** Signaling inhibition results in increased BBB permeability allowing for improved chemotherapy delivery.

WNT inhibition holds potential to target not only the tumor itself, but also associated vasculature. As we have discussed, there is some evidence for the role of WNT signaling in pathologic, and even tumor angiogenesis. Even more intriguingly, WNT inhibition of glioma cells and influence of microvasculature, could target the BBB, essentially attempting to replicate the vascular phenotype of WNT medulloblastoma. This could significantly increase the fraction of systemically administered temozolomide that penetrates the CNS, which is normally only 20% of the systemic concentration (Ostermann et al., 2004; Portnow et al., 2009). A variety of systemically administered agents have been evaluated to elicit increased BBB permeability (Jackson et al., 2016, 2017). However, none have provided a sizeable and sustained therapeutic effect. Prospective clinical trials with WNT inhibition and concomitant chemoradiotherapy will help determine the therapeutic benefit of WNT inhibition in patients with malignant gliomas. Such studies will provide the opportunity to evaluate not only clinial outcomes, but also the effects of WNT inhibition on tumor angiogenesis and on the BBB in relation to drug delivery (Figure 2).

The WNT pathway is an intricate and ubiquitous signaling cascade which influences many processes in health and disease. Interestingly, it plays key roles in the pathogenesis of multiple types of brain tumors. The WNT signaling pathway is of great value in glioblastoma research because it represents a potential means of targeting an aggressive, highly vascularized tumor by impacting glioma stem cells, tumor invasiveness, therapeutic resistance, angiogenesis, and drug delivery in an effort to improve overall survival.

## Author contributions

MM extensively researched the subject area to craft a paper detailing the role of WNT and malignant glioma development/proliferation. YM and MG provided insight into paper content and structure with their background in vascular biology/development and clinical neuro-oncology, respectively. SJ is the principal investigator on this review paper and provided intellectual input regarding content, paper structure and flow of manuscript with background as a molecular biologist/neuro-oncologist.

### Conflict of interest statement

The authors declare that the research was conducted in the absence of any commercial or financial relationships that could be construed as a potential conflict of interest.
